# 471. Descriptive Analysis of Multiinflammatory Syndrome in Children (MIS-C) Secondary to COVID 19 Infection in a Predominantly Latino Population in Western Massachusetts

**DOI:** 10.1093/ofid/ofab466.670

**Published:** 2021-12-04

**Authors:** Julieta Rodriguez, Nicholas Karr, Grzegorz Danielczok, Donna J Fisher, Ingrid Y Camelo

**Affiliations:** 1 UMMS Baystate, Springfield, Massachusetts; 2 Baystate Medical Center, Springfield, Massachusetts; 3 UMMS - Baystate Medical Center, Northampton, Massachusetts; 4 Baystate Medical Center/Baystate Children’s Hospital, Longmeadow, Massachusetts; 5 UMass- Baystate Medical Center/Baystate Children’s Hospital, Northampton, Massachusetts

## Abstract

**Background:**

COVID-19 infection is usually mild in children. Progression to severe disease with multiple organ systems compromise described as Multi Inflammatory Syndrome in Children (MIS-C) is a rare occurrence believed to be immunologically mediated. Previous reports describe a possible link between children of Latino origin and high incidence of MIS-C in the US. 40% of the total population in Western Massachusetts is of Latino origin.

**Methods:**

Retrospective chart review of 30 children admitted to Baystate Medical Center in Springfield, Massachusetts from April 2020-June 2021 meeting Centers for Disease Control and Prevention (CDC) criteria for MIS-C. Demographics, laboratory data, and clinical outcomes including progression to Macrophage Activation Syndrome (MAS) were analyzed.

**Results:**

60% of children were Hispanic. Mean age (9.1 yrs). Range (3m-20 yrs). COVID PCR positive (78%) and COVID Antibody positive (68%). The most common symptom was fever (96.8%) followed by gastrointestinal symptoms (84%). Respiratory symptoms (29%), dermatological manifestations (39%). Most common comorbidity, asthma (19%) followed by obesity (17%). Leukocytosis (47%), lymphopenia (45%), Anemia (55%), thrombocytopenia (20%), high CRP (90%), ferritinemia (57%), acute kidney injury (20%), elevated liver enzymes (53%), 52% children had electrocardiogram (EKG) abnormalities, 34% had abnormal echocardiograms, none displayed coronary artery dilation. Progression to MAS (20%). All patients were treated with intravenous immunoglobulin G, steroids, aspirin, and anakinra (IL1 receptor antagonist) if progression to MAS. All patients survived.

**Conclusion:**

In our population, gastrointestinal symptoms were predominant despite a high prevalence of asthma and obesity, previous reports of children with MIS-C describe predominance of respiratory manifestations. We did not encounter any coronary aneurysms during admission. Most children had positive PCR or Antibodies for COVID 19 and showed important abnormalities in multiple cell lines and inflammatory markers. More research is needed to fully understand ethnical risk factors associated with disease severity especially the risk of progression to MAS from MIS-C in children of Latino origin diagnosed with COVID 19 infection.

**Disclosures:**

**All Authors**: No reported disclosures

